# Percolation-Induced Ferrimagnetism from Vacancy Order in [Gua]Mn_1–*x*_Fe_2*x*/3_(HCOO)_3_ Hybrid Perovskites

**DOI:** 10.1021/jacs.4c03407

**Published:** 2024-05-09

**Authors:** Johnathan M. Bulled, Alexandra Willis, Zoé Faure Beaulieu, Simon J. Cassidy, Jonas Bruckmoser, Hanna L. B. Boström, Andrew L. Goodwin

**Affiliations:** † Department of Chemistry, University of Oxford, Inorganic Chemistry Laboratory, Oxford OX1 3QR, United Kingdom; ‡ Department of Chemistry, Technical University of Munich, Lichtenbergstraße 4, 85748 Garching, Germany; § Wallenberg Initiative Materials Science for Sustainability, Department of Materials and Environmental Chemistry, Stockholm University, SE-114 18 Stockholm, Sweden; ∥ Department of Materials and Environmental Chemistry, Stockholm University, SE-114 18 Stockholm, Sweden

## Abstract

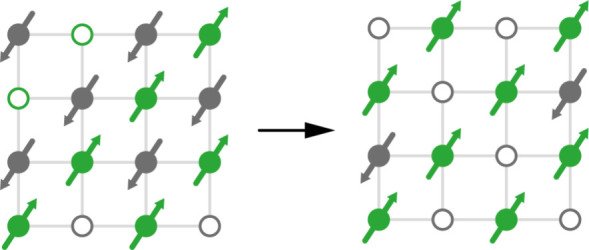

We report the magnetic behavior of the hybrid perovskites [Gua]Mn_1–*x*_Fe_2*x*/3_□_*x*/3_(HCOO)_3_ (0 ≤ *x* ≤ 0.88), showing that vacancy ordering drives bulk ferrimagnetism for *x* > 0.6. The behavior is rationalized in terms of a simple microscopic model of percolation-induced ferrimagnetism. Monte Carlo simulations driven by this model reproduce the experimental dependence of magnetic susceptibility on *x* and show that, at intermediate compositions, domains of short-range vacancy order lead to the emergence of local magnetization. Our results open up a new avenue for the design of multiferroic hybrid perovskites.

Molecular perovskites are a family of framework materials with an ABX_3_ stoichiometry and a simple cubic network structure. They can incorporate molecular (A) and transition-metal (B) cations as well as molecular anions (X), thereby allowing for the design of materials which combine the properties of their constituent parts.^1−3^ The formate perovskites (X = HCOO^–^) hold particular currency as candidate multiferroic materials which simultaneously host magnetic and electric polarization^4−6^ for applications in sensors and information storage devices.^7,8^ Effective design of multiferroics in conventional inorganic perovskites is difficult because of the incompatible electronic structure design criteria for polarization (closed-shell species) and magnetism (open-shell species).^9^ In the hybrid perovskites, the combination of molecular and orbital degrees of freedom gives a large number of strategies for inducing ferroelectricity.^10−15^ Less clear, however, is how to generate hybrid perovskites with strong bulk magnetic polarization: nearly all hybrid perovskites are canted antiferromagnets with only weak moments that result from antisymmetric interactions.^10,16,17^ An obvious challenge, therefore, is to devise new routes to enhance bulk magnetization in hybrid perovskites.

It was in this context that we developed an interest in the strategy of percolation-induced ferrimagnetism (PIF), whereby occupational ordering of a nonmagnetic dopant in an antiferromagnet leads to an imbalance between sublattice magnetizations, and hence ferrimagnetism (Figure 1a).^18^ This is the mechanism at play in, e.g., the series Fe_1–*x*_Zn_*x*_F_2_, which shows a crossover from antiferromagnetism to ferrimagnetism as *x* is increased above a critical percolation threshold (*x*_p_ ≃ 0.86 in this case).^18−20^ It is also related to the mechanism that drives relaxor ferromagnetism in La_3_Ni_2_SbO_9_.^21^ In order for solid solutions to host PIF, the nonmagnetic and antiferromagnetic magnetic constituents cannot be statistically distributed on the cation sublattice but must instead obey local ordering rules that drive partitioning above some critical concentration, the value of which is characteristic of the sublattice geometry. The difference in Fe^2+^/Zn^2+^ ionic radii provides such a cation ordering mechanism in Fe_1–*x*_Zn_*x*_F_2_.^19^

**Figure 1:**
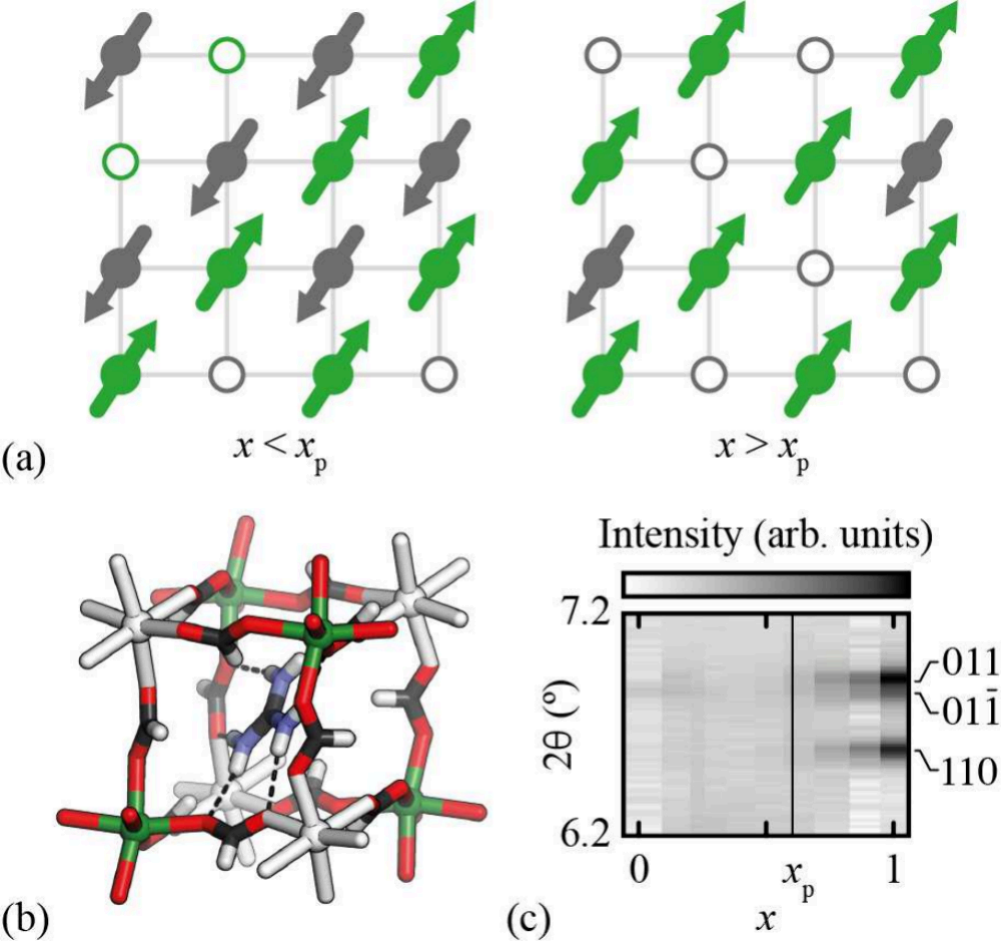
(a) Schematic of the PIF mechanism in dilute antiferromagnets. If nonmagnetic dopants (e.g., vacancies, shown as open circles) are forbidden from occupying neighboring sites, then at doping levels *x* > *x*_p_ they are distributed preferentially on a single sublattice (here, colored gray). The resulting imbalance in sublattice magnetization gives rise to a net ferrimagnetic moment. (b) Representation of the vacancy ordered structure of [Gua]Mn_1–*x*_Fe_2*x*/3_□_*x*/3_(HCOO)_3_, with cation-rich and cation-poor B sites colored green and white, respectively. (c) A film plot of X-ray powder diffraction patterns (λ = 0.8248300(1) Å) showing the symmetry breaking associated with vacancy order at *x* ≥ *x*_p_: the 011/011̅ reflection pair split and the 110 reflection, ordinarily forbidden, appears. Data taken from ref (22).

The solid solution [Gua]Mn_1–*x*_Fe_2*x*/3_□_*x*/3_(HCOO)_3_ (Gua = C(NH_2_)_3_^+^) is an interesting family in this respect because of the emergence of vacancy order at sufficiently large values of *x*. In this system, the B site of the perovskite structure is jointly occupied by Mn^2+^, Fe^3+^, and vacancies (□).^22^ The two cations share the same electronic configuration and are therefore similar magnetically (μ_eff_^Fe(III)^ = μ_eff_^Mn(II)^ ≃ 5.91 μ_B_); the vacancies play the role of nonmagnetic dopants. It is thought that the strong hydrogen-bonding interactions between guanidinium cations and formate anions are particularly important in stabilizing vacancies in this system.^22^ Anticlustering of vacancies leads to checkerboard vacancy order beyond a percolation threshold *x* ≥ *x*_p_ ≃ 0.6, as evidenced by the emergence of sharp superlattice reflections and accompanying symmetry lowering (Figure 1b,c); the crystallographic details of this transition are discussed in ref (22). The vacancy-ordered phase is conceptually related to the double perovskite structure, in which the B-site occupancy alternates on two face-centered cubic sublattices. Here, this B-site occupancy alternates between vacancy-rich (magnetically poor) and vacancy-poor (magnetically rich) sites, and so this imbalance of magnetic and nonmagnetic species suggests that [Gua]Mn_1–*x*_Fe_2*x*/3_□_*x*/3_(HCOO)_3_ may in principle satisfy the design criteria for PIF.

To test for the effect of vacancy order on magnetic order in this family, we carried out magnetometry measurements for the nine compositions investigated in ref (22). The vacancy-free (*x* = 0) end member behaves as a typical antiferromagnet, as indicated by a negative Curie–Weiss temperature (θ_CW_ = −12.2(5) K, see SI). The zero-field-cooled (ZFC) magnetic susceptibility shows a maximum at *T*_c_ = 8.80(5) K, below which it diverges from the field-cooled (FC) susceptibility (Figure 2a); this behavior is characteristic of the development of long-range antiferromagnetic order. We also observe a small canted moment within the ordered phase (8(3) emu mol^–1^ at 2 K) evident in the isothermal magnetization measurement. Collectively, these various observations are typical of the canted antiferromagnetism of other Mn^2+^-containing formate perovskites studied elsewhere.^23−25^ As the vacancy fraction increases across the series, the corresponding trends in magnetic susceptibility change. The maximum in ZFC susceptibility at *T*_c_ shifts to higher temperature and gradually disappears as the low-temperature susceptibility starts to diverge (Figure 2a). In all cases, antiferromagnetic interactions dominate: the Curie–Weiss temperature remains negative, and its magnitude increases slightly as Mn^2+^ is replaced by Fe^3+^ (see SI). Yet a hysteresis loop opens in the magnetization curves at high vacancy concentrations, with a maximum residual moment of 113(5) emu mol^–1^ for the *x* = 0.88 sample (Figure 2b). All of these observations are consistent with a transition from canted antiferromagnetism to ferrimagnetism as vacancy order develops.

**Figure 2:**
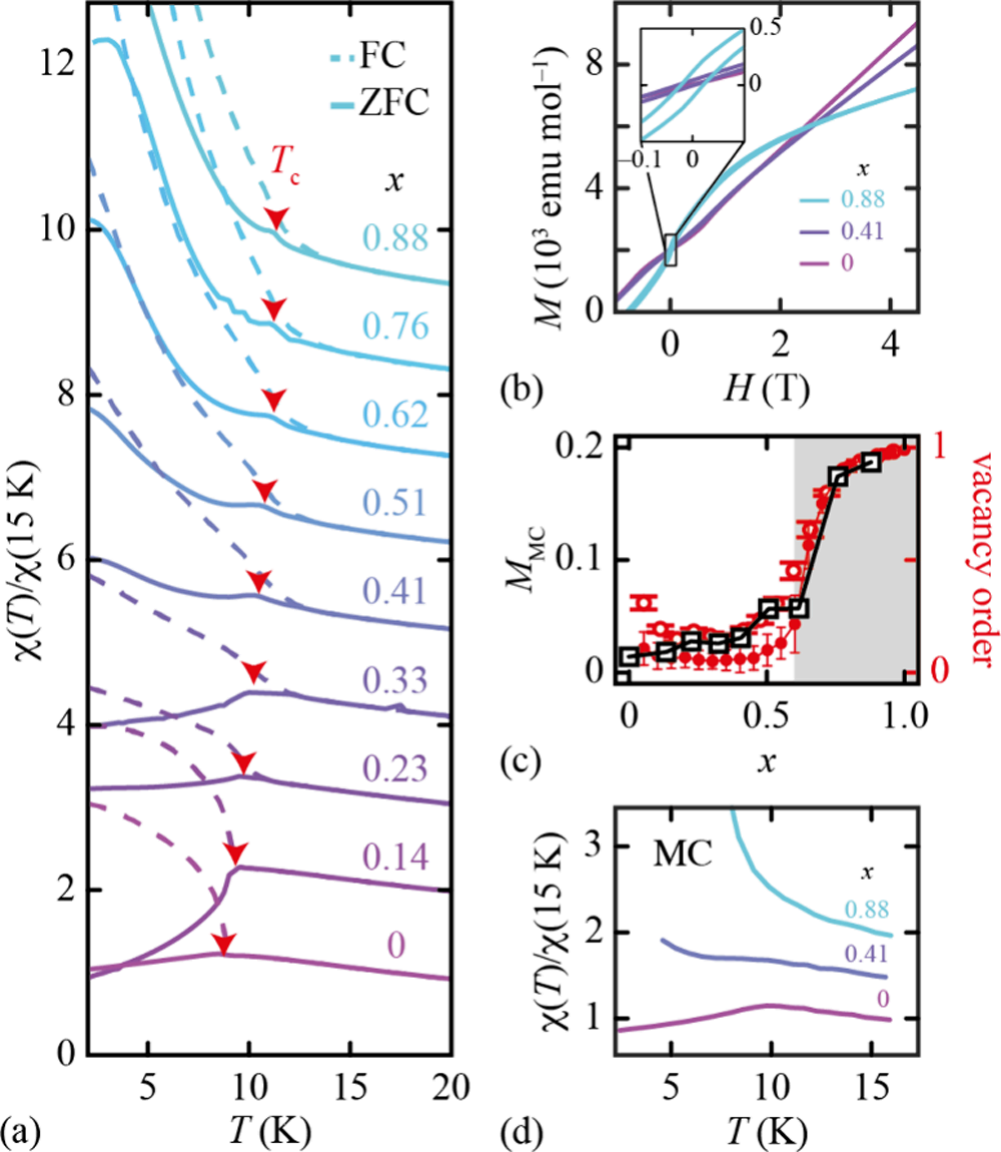
(a) Field-cooled (dashed colored lines) and zero-field-cooled (solid colored lines) magnetic susceptibility (*H* = 100 Oe) as a function of temperature for nine members of the series [Gua]Mn_1–*x*_Fe_2*x*/3_(HCOO)_3_. To aid comparison, each data set is normalized to its value at 15 K, and data for successive compositions are offset by one unit. Magnetic ordering temperatures are indicated by red arrows. (b) Magnetization curves at 2 K shown for three representative compositions. The inset highlights the opening of a hysteresis loop at high vacancy fractions. (c) Calculation of the low temperature limit of the magnetization for each member of the series (0 ≤ *x* ≤ 0.88) studied (black) from the MC model described in the text, compared with the long-range vacancy order parameter determined in our MC simulations (open red circles) and taken from ref (22) (filled red circles). The PIF regime is shaded gray. (d) Magnetic susceptibilities for three key compositions as generated from the MC procedure described in the text. Successive data sets are offset by 0.5 units for clarity.

In order to better understand the trends in our magnetometry data, we used Monte Carlo (MC) simulations performed in two stages. The first stage involved determining, for each composition *x*, an appropriate vacancy distribution; we achieved this using the same nearest-neighbor vacancy-avoidance model developed in ref (22). These configurations were then used for a subsequent spin-MC simulation, where the nonvacant sites were decorated with Heisenberg spins **S**_*i*_ and the MC energy was given by
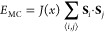
1Here, the sum is taken over pairs of neighboring magnetic (nonvacant) sites, and we are approximating magnetic interactions using the very simplest isotropic Heisenberg model. The coupling constant *J*(*x*) was parametrized to reproduce the experimental dependence of the magnetic transition temperature on *x*, varying linearly from 6 to 9 K as *x* increases (see SI for further discussion).

Key results from these MC simulations are shown in Figure 2. Vacancy order develops as expected for *x* ≥ *x*_p_ ≃ 0.6, and crucially this ordering couples to an abrupt increase in the low temperature magnetization *M*_MC_, signifying the onset of PIF (Figure 2c). The value of *M*_MC_ was estimated via simulated annealing to a low temperature (*T*_MC_ = 0.2 *J*; see SI for further details). The fluctuations in our MC simulations also allowed us to calculate the magnetic susceptibility, as shown in Figure 2d. We find excellent qualitative agreement in reproducing the key trends identified in our earlier discussion, including the evolution from antiferromagnetism to ferrimagnetism associated with PIF. A comparison for each member of the series is included in the SI.

While we only expect global PIF at values of *x* > *x*_p_, both the model and experiment show some enhancement of the low-temperature susceptibility at vacancy concentrations below the percolation threshold, where any vacancy order present is only short-range in nature. We explored this regime in greater detail by extending our MC simulations to larger supercells of sufficient size to include multiple domains of local vacancy order. The low-temperature magnetic structures resulting from these simulations are shown in Figure 3a for three compositions spanning the percolation threshold. The spin configurations themselves are relatively simple in that, locally, neighboring spins point in approximately opposite directions. The more complex interplay with vacancy distributions can be seen by visualizing local vacancy order and magnetization (Figure 3b,c), each calculated using the approach of ref (26), as detailed in the SI. At high vacancy fractions (*x* = 0.9), vacancy order percolates but antiphase domains persist—these are regions in which the choice of vacancy-rich/magnetically poor fcc sublattice differs from that of the bulk. This switch in vacancy-order phase translates to a switch in direction of the corresponding magnetization, such that the presence of these domains would affect the magnitude of the ordered moment. Recent developments in high-resolution magnetic imaging methods, such as magnetic force microscopy (MFM) or X-ray microscopy, might even allow for the direct imaging of this domain structure.^27−29^ At vacancy fractions close to the percolation limit (*x* = 0.6), there are strong fluctuations in vacancy order that correlate with domains in the local magnetization. And at low vacancy fractions (*x* = 0.3), these regions of local magnetization are still present, albeit that they are smaller and magnetically weaker. Given the experimental observation of hysteresis loop opening in the *x* = 0.41 sample (Figure 2b), we suggest that the existence of local vacancy order might lead to an imbalance between magnetization within small magnetic domains for *x* < *x*_p_. This interpretation could help explain why the transition from antiferromagnet to ferrimagnet is more gradual in practice than the PIF picture might suggest at face value.

**Figure 3:**
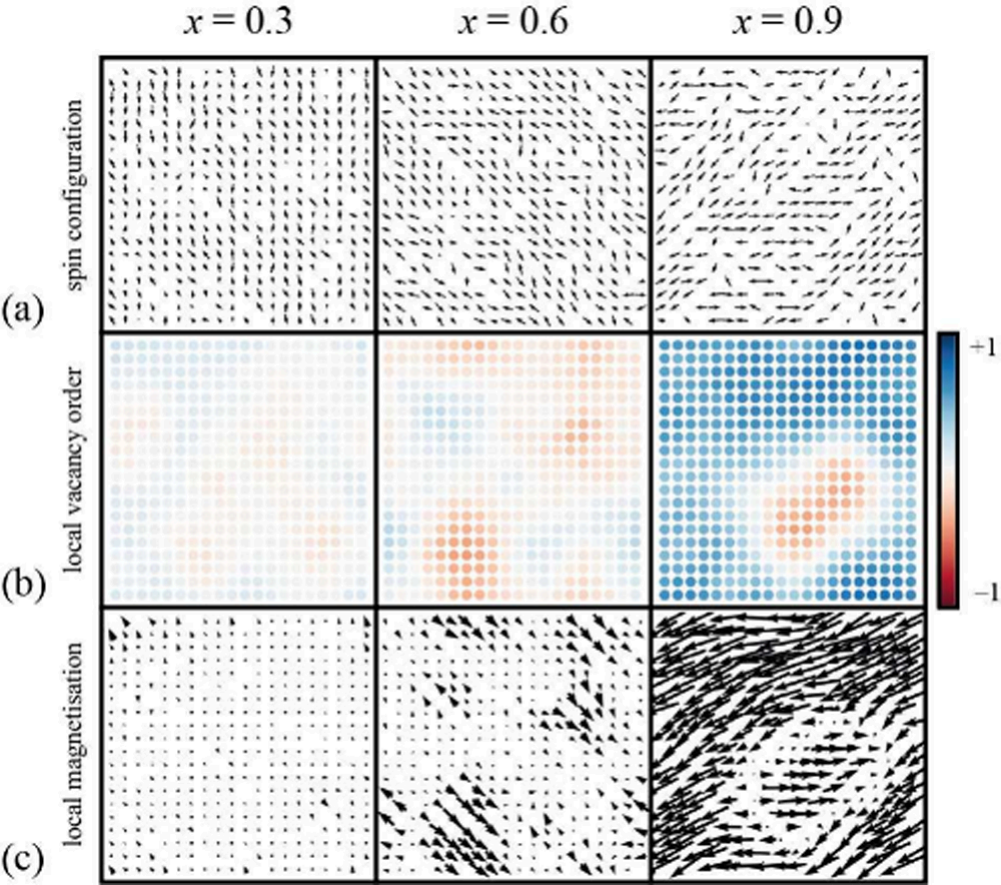
(a) Direct examination of domain structure in MC configurations, showing one plane of a 20 × 20 × 20 simulation at three vacancy concentrations, spanning the percolation limit. The (b) local vacancy and (c) local magnetic order parameters show the domain structure that develops in the simulations. Note the correlation between the regions of vacancy and magnetic order.

The closely related series [CH_3_NH_3_]Mn_*x*_Zn_1–*x*_(HCOO)_3_, whose structure and magnetism were investigated in ref (30), provides a useful comparison for our analysis. In that family, magnetic dilution tunes the nature of the magnetic order but does not give rise to PIF because there is no long-range order of the Mn^2+^ and Zn^2+^ ions. Intriguingly, the mixed-metal formates [Gua]Cu_*x*_M_1–*x*_(HCOO)_3_ (M = Mn, Zn, Mg) all appear to show short-range cation order, but the tendency in those systems is for like cations to cluster.^31^ Such ordering is also incompatible with PIF.

It follows that a key ingredient for realizing PIF in formate perovskites is the checkerboard vacancy order, which we drive by aliovalent doping in the case of [Gua]Mn_1–*x*_Fe_2*x*/3_(HCOO)_3_. In this system, both cations had the same electron configuration, and there was no evidence of long-range order of the cations. However, neither condition is essential for the physics. Thinking forward to combining the emergence of bulk magnetization with electric polarization in related systems, one strategy will be to exploit the combination of cooperative A-site orientational order and B-site Jahn–Teller order in breaking inversion symmetry.^10,12,32^ An obvious family worthy of exploration, if its members can be prepared, is [Gua]Cu_1–*x*_Fe_2*x*/3_(HCOO)_3_: one end member (*x* = 0) is polar,^10,32,33^ and we now know the other (*x* = 1) to be a ferrimagnet. What happens in between? While in principle there are a number of other formate perovskite families that exhibit polar phases, we anticipate that the use of guanidinium as an A-site cation may be key for PIF because its strong hydrogen bonding is implicated in stabilizing B-site vacancies.^22,34^

One important consideration in developing new PIF formate perovskites—relevant in particular to Cu^2+^-containing systems—will be whether the simple isotropic Heisenberg model we have used here remains a useful approximation. Even in the case of [Gua]Mn_1–*x*_Fe_2*x*/3_(HCOO)_3_, there are signs that more complex physics is required to capture the full magnetic behavior: a spin-flop transition reflects the presence of magnetic anisotropy, and a small residual magnetic moment in the measured isothermal magnetization curves indicates antisymmetric interactions are also involved (see SI for further discussion). Fortunately, we find that neither greatly affects the PIF mechanism at play. But the strongly directional nature of magnetic interactions in Cu^2+^ systems, itself a consequence of uneven occupancy of e_g_^*^ orbitals in d^9^ configurations, will likely complicate matters considerably—even if one ignores the (likely) possibility of interplay between vacancy distributions and orbital order. Such cases may lead to stoichiometry-dependent anisotropy of the kind seen in [Zn_1–*x*_Ni_*x*_(HF_2_)(pyz)_2_]SbF_6_, for example.^35^ Whatever emerges, our key result here is to have shown how simple chemical modification of a hybrid perovskite can have a profound effect on its magnetic behavior and hence open new avenues for functional materials design that may also be relevant to other inorganic and hybrid perovskite families (such as the Prussian blue analogues) supporting correlated vacancy order.^36^

## Supporting Information Available

Details regarding synthesis, magnetometry, Monte Carlo simulations, and parametrization of *J*(*x*) in eq 1; extended version of Figure 2, showing additional values of *x* (PDF)
