# Supplementary material for: Percolation-Induced Ferrimagnetism from Vacancy Order in [Gua]Mn1–xFe2x/3(HCOO)3 Hybrid Perovskites
Source: J Am Chem Soc. 2024 May 9;146(20):13714–8. doi: 10.1021/jacs.4c03407 (PMC11117395; doi:10.1021/jacs.4c03407)
Supplement: Supplementary file 1 — ja4c03407_si_001.pdf [file ja4c03407_si_001.pdf]

Percolation-induced ferrimagnetism from vacancy order in $[Gua]Mn_{1-x}Fe_{2x/3}(HCOO)_3$ hybrid perovskites:

SUPPLEMENTARY INFORMATION

Jonathan. M. Bulled,[a] Alexandra Willis,[a] Zoe Faure Beaulieu,[a] Simon J. Cassidy,[a] Jonas Bruckmoser,[b] Hanna L. B. Boström,[a,c,d] and Andrew L. Goodwin[a*]

[a]Department of Chemistry, University of Oxford, Inorganic Chemistry Laboratory, Oxford, OX1 3QR, U.K.

[b]Department of Chemistry, Technical University of Munich, Lichtenbergstraße 4, Garching, Germany

[c]Wallenberg Initiative Materials Science for Sustainability, Department of Materials and Environmental Chemistry, Stockholm University, SE-114 18, Stockholm, Sweden

[d]Department of Materials and Environmental Chemistry, Stockholm University, SE-114 18, Stockholm, Sweden

*To whom correspondence should be addressed;
E-mail: andrew.goodwin@chem.ox.ac.uk

Contents

1 Synthesis **2**

2 Magnetometery **3**

3 Monte Carlo Model and Parameterisation **8**

4 References **15**

1 Synthesis

Polycrystalline samples of the composition [Gua]$Mn_{1-x}Fe_{2x/3}(HCOO)_3$ ($x = 0$, 0.14, 0.23, 0.33, 0.41, 0.51, 0.62, 0.76, 0.88) were synthesised by combining solutions of guanidinium carbonate, formic acid, and stoichiometric mixtures of manganese(II) nitrate and iron(III) chloride. The solid product was isolated by filtration and washed. The details of this synthesis are reported in Ref. 1, alongside characterisation of the samples used in this study by atomic adsorption spectroscopy and X-ray powder diffraction.

2 Magnetometery

Low field measurements

We measured the field-cooled (FC) and zero-field-cooled (ZFC) magnetometry on warming from 2 to 300 K on an MPMS-3 magnetometer on $\sim$ 10 mg portions of 9 members of the series investigated in Ref. 1 with an applied field of 100 Oe. A heating rate of $\sim$ 6 min K^{-1} was used over the temperature range $2 \leq T < 20$ K and $\sim$ 0.33 min K^{-1} over the temperature range $20 < T < 300$ K.

The ZFC and FC magnetometery data are plotted in Figure S1(a) for each member of the series. The data are normalised by the mass of the sample and the relative formula mass of [Gua]$Mn_{1-x}Fe_{2x/3}(HCOO)_3$. The two curves bifurcate a little above T_c, a temperature which is extracted from a discontinuity in the gradients. The value of T_c and its uncertainty are plotted as a function of composition in Figure S1(b).

Curie-Weiss

From the zero field susceptibility, we extract the Curie-Weiss fit parameters ($\theta_{\rm CW}, C$) according to

$$(\theta_{\rm CW}, C) = \underset{\theta_{\rm CW}, C}{\arg\min} \sum_i \left(\frac{1}{\chi_i} - \frac{C}{T_i - \theta_{\rm CW}} \right)^2 \quad (1)$$

where the sum is performed over the temperature range $50 < T < 300$ K. The lower temperature chosen here is the main source of error in this value. Alternative temperature ranges $30 < T < 300$ K and $100 < T < 300$ K are calculated to estimate the error inherent to this fitting procedure. The inverse susceptibility is plotted alongside the extracted Curie-Weiss fits in Figure S2(a), showing that each fit is satisfactory, with error bounds given by the $30 < T < 300$ K and $100 < T < 300$ K fits.

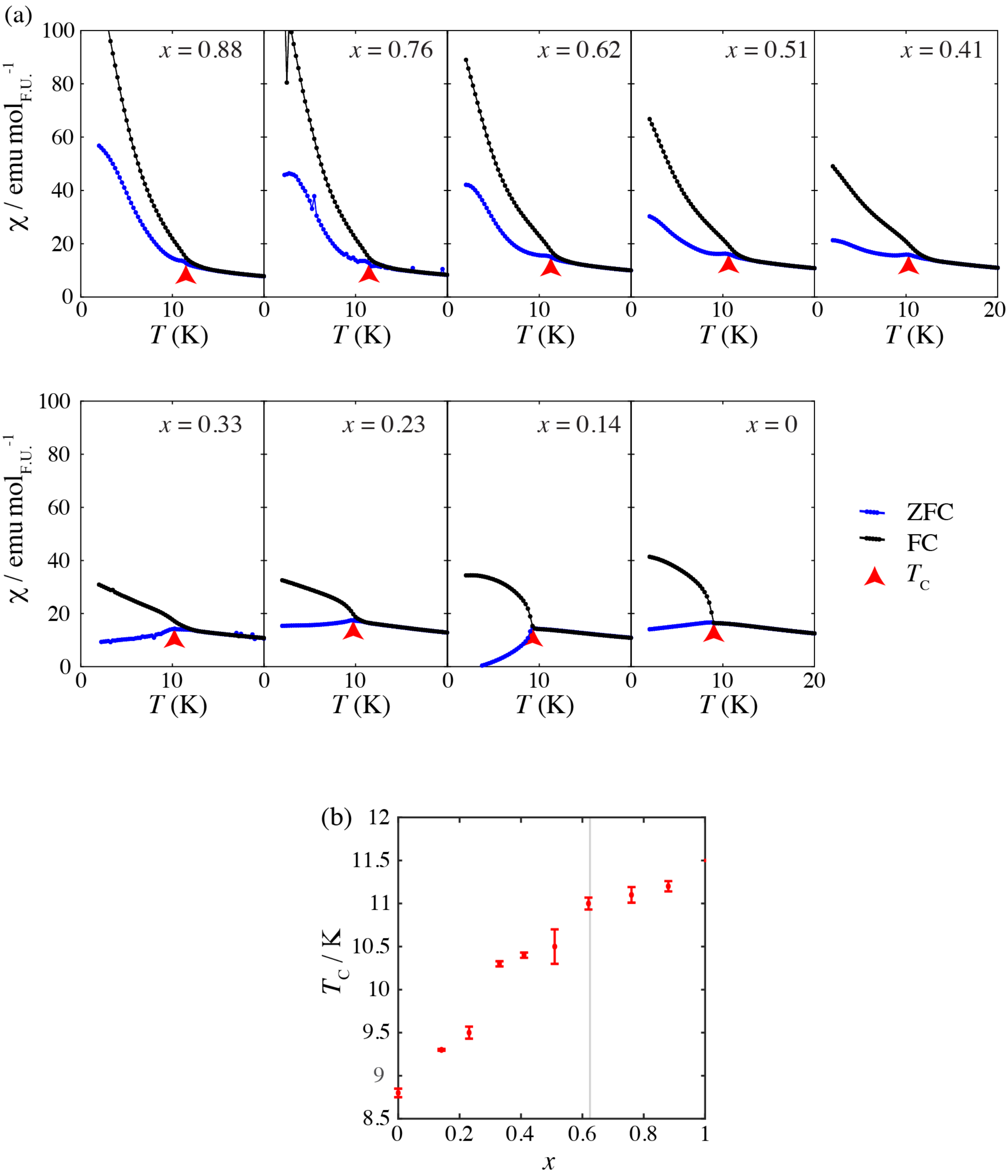

Figure S1: (a) Zero field cooled (blue) and field cooled (black) measurements, plotted across the series, showing their bifurcation at (or just above) T_c and the change of the behaviour around the maximum at $x \sim x_p$. Each value is normalised per mol of formula units (F.U.s). The value of T_c (red arrow) is estimated from the maximal feature in the ZFC susceptibility, with its error coming from our ability to extract the value. (b) The variation of T_c with x, showing a smooth compositional and no abrupt change at x_p.

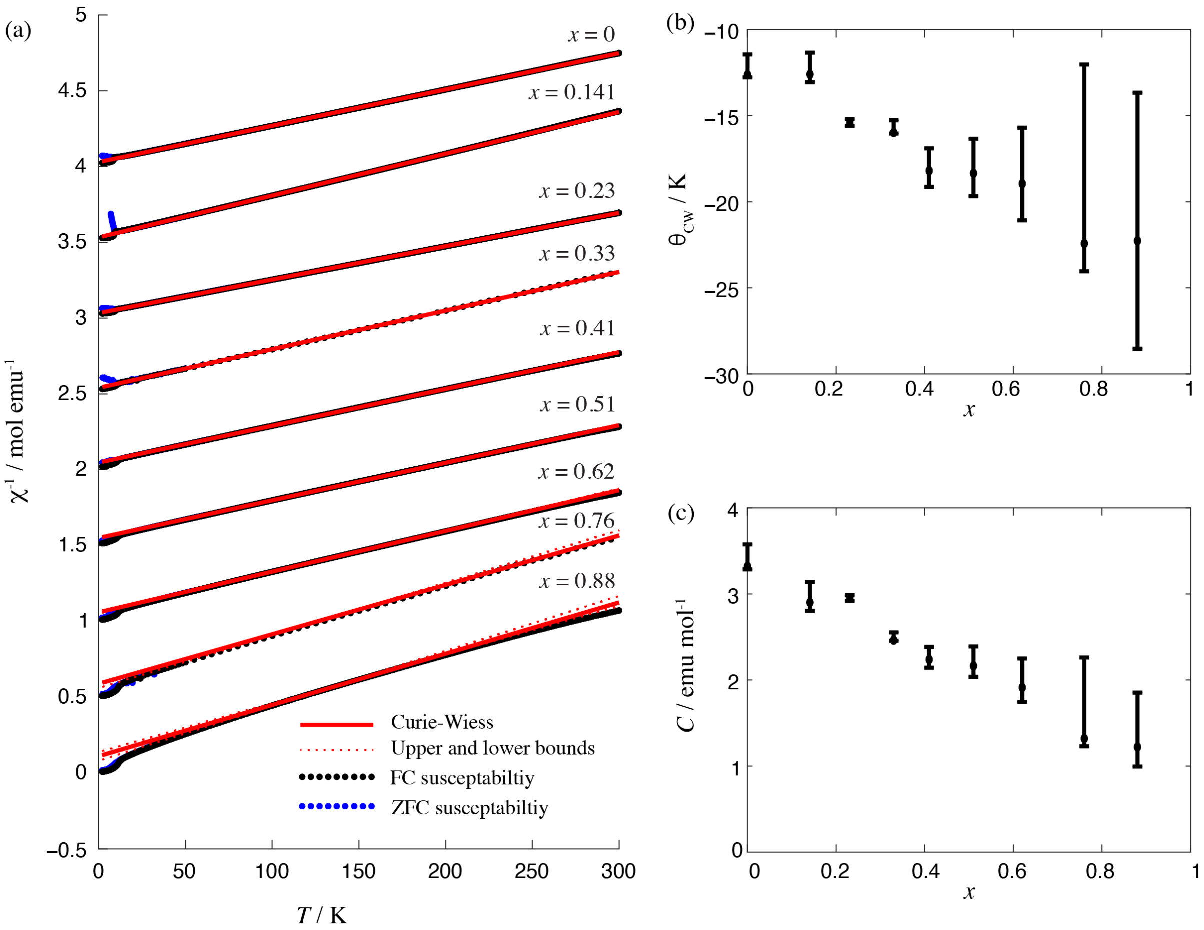

Figure S2: (a) Inverse susceptibility, extracted from zero field cooled (blue) and field cooled (black) measurements, plotted across the series compared with their Curie-Weiss fit. Each successive curve is offset by 0.5 units. (b,c) The dependence of θ_{CW} and C (respectively) on x, as extracted from a fit to the data over the range $50 < T < 300$ K. The error bars in this plot are estimated from the change in fitted value as the minimum fitting temperature is varied between 10 and 100 K.

Variable Field Measurements and Hysteresis

Variable field magnetometry measurements were carried out using the same device for each member of the series, showing a marked change in the magnetic behaviour across the series. Magnetic field was cycled though an $H = 0 \to 5 \to -5 \to 0\,\mathrm{T}$ loop in order to understand the degree of magnetic hysteresis. The results are plotted in Figure S3(a), and shown as their gradient in Figure S3(c). The residual magnetisation [Fig S3(b)] is extracted as the average of residual magnetisation on the first and second demagnetisations—*i.e.* the y-intercept of the magnetisation on the application and removal of field. Stated explicitly,

$$M_{\mathrm{res}} = \frac{1}{2}\left[M(H = 0 \to +5 \to 0) - M(H = 0 \to +5 \to -5 \to 0)\right] \tag{2}$$

and the error on this value is estimated by

$$\Delta M_{\mathrm{res}} = \frac{1}{\sqrt{2}}\left[M(H = 0 \to +5 \to 0) + M(H = 0 \to +5 \to -5 \to 0)\right]. \tag{3}$$

On doping with Fe^{3+}, a magnetic hysteresis loop is opened at low field. At $x = 0$, there is a very small residual magnetisation of $M_{\mathrm{res}} = 6 \times 10^{-4}$ emu mol^{-1}, consistent with the values seen in manganese formate perovskites[S2–4], but this value is enhanced by more than an order of magnitude to a maximum value of 1.5×10^{-2} emu mol^{-1} for the $x = 0.88$ member of the series. The value of the residual magnetisation is extracted as the average of the value on magnetisation and demagnetisation. Since there are only two averages contributing to this value in equations (2) and (3), our uncertainty on the value of M_{res} is large, but when $x > x_{\mathrm{p}}$, the value clearly rises above the level of this uncertainty.

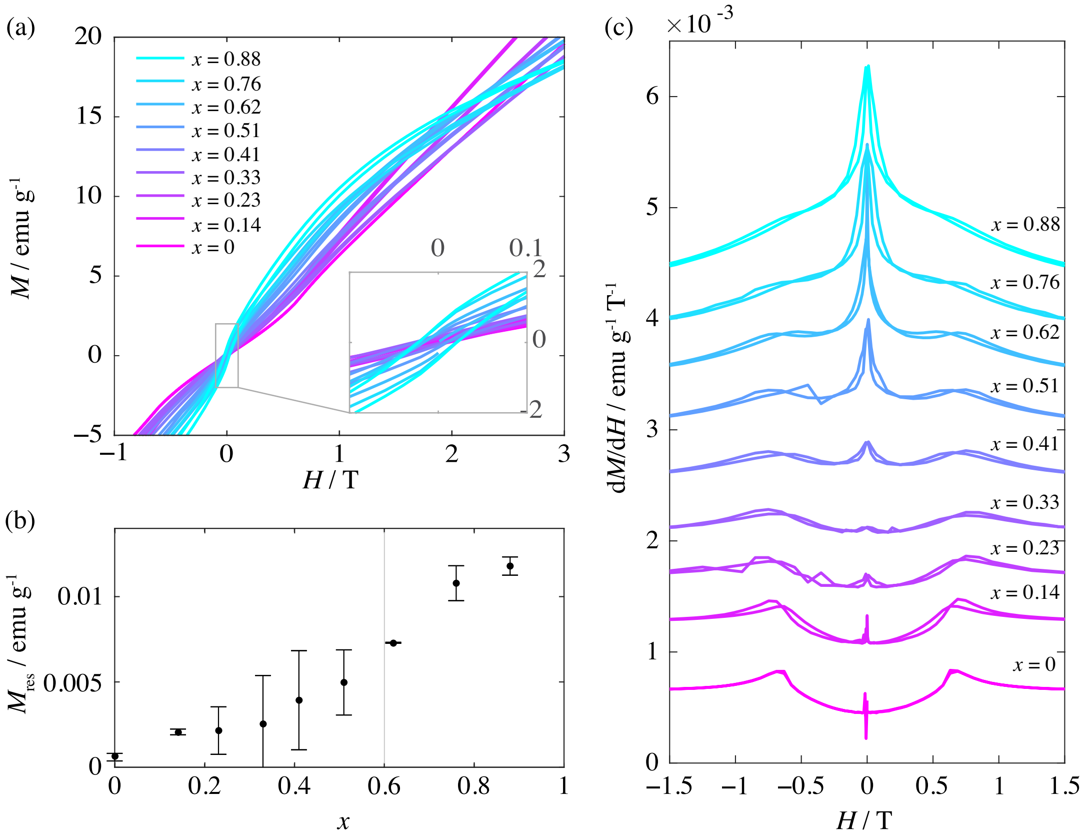

Figure S3: (a) Applied field measurements measured with the magnetisation cycle described in the text. The hysteresis loop, which opens for larger values of x is highlighted in the inset. (b) The dependence of remanent magnetisation on x. (c) The gradient of the magnetisation. For clarity, each successive curve is offset by 0.5 units.

3 Monte Carlo Model and Parameterisation

Overview

In order to better understand the changes to the magnetisation at finite temperature and intermediate vacancy concentration, we built on on the previous Monte Carlo simulations which explained the vacancy correlations in the system[S1]. These simulations were able to reproduce the ordering transition at the correct percolation threshold. The general simulation approach is shown schematically in Figure S4(a). First, vacancies are randomly distributed in a supercell; then the first MC procedure redistributes the vacancies such that there are no nearest-neighbour contacts. In the next series of Monte Carlo simulations, magnetic moments are allocated to the sites and evolved according to the spin Hamiltonian at finite temperature. Here, we detail these steps and describe the procedure used to parameterise the Hamiltonian.

Vacancy MC

The bulk of the MC simulations were performed on $6 \times 6 \times 6$ supercells of the parent unit-cell, consistent with the work of Ref. 1. Subsequent simulations detailed in section 3 to analyse the domain structure were carried out on larger $20 \times 20 \times 20$ supercells—large enough to potentially host multiple domains.

In previous work on the system[S1], the ordering of vacancies was demonstrated using a simplified cubic model (which neglects the differences between the three crystallographically-inequivalent directions). In this way, the vacancy distribution of the system can be described as arising from vacancy anti-clustering (i.e. the tendency for vacancies to avoid nearest-neighbour contacts). The first step of our simulation reproduces these established results. Vacancies are randomly distributed in the unit cell and simulated annealing is used to produce a set of maximum entropy configurations which minimise the number of vacancy–vacancy nearest-neighbours. This process is repeated for many values in the range $0 \leq x \leq 1$, chosen to correspond to the experimental values of x discussed above. Further simulations were subsequently carried out on finer x grids and over a larger range.

By tracking an order parameter Boström and coworkers determined the onset of anticlustering to be $x \approx 0.6$[S1]. The order parameter for the chequerboard ordering at the vector $\mathbf{k} = \left[\frac{1}{2}, \frac{1}{2}, \frac{1}{2}\right]$ is defined as

$$\alpha(x) = \frac{1}{N_{\text{vac}}} \left| \sum_{i \in \text{sites}} o_i \exp 2\pi \mathrm{i} \mathbf{r}_i \cdot \mathbf{k} \right|, \tag{4}$$

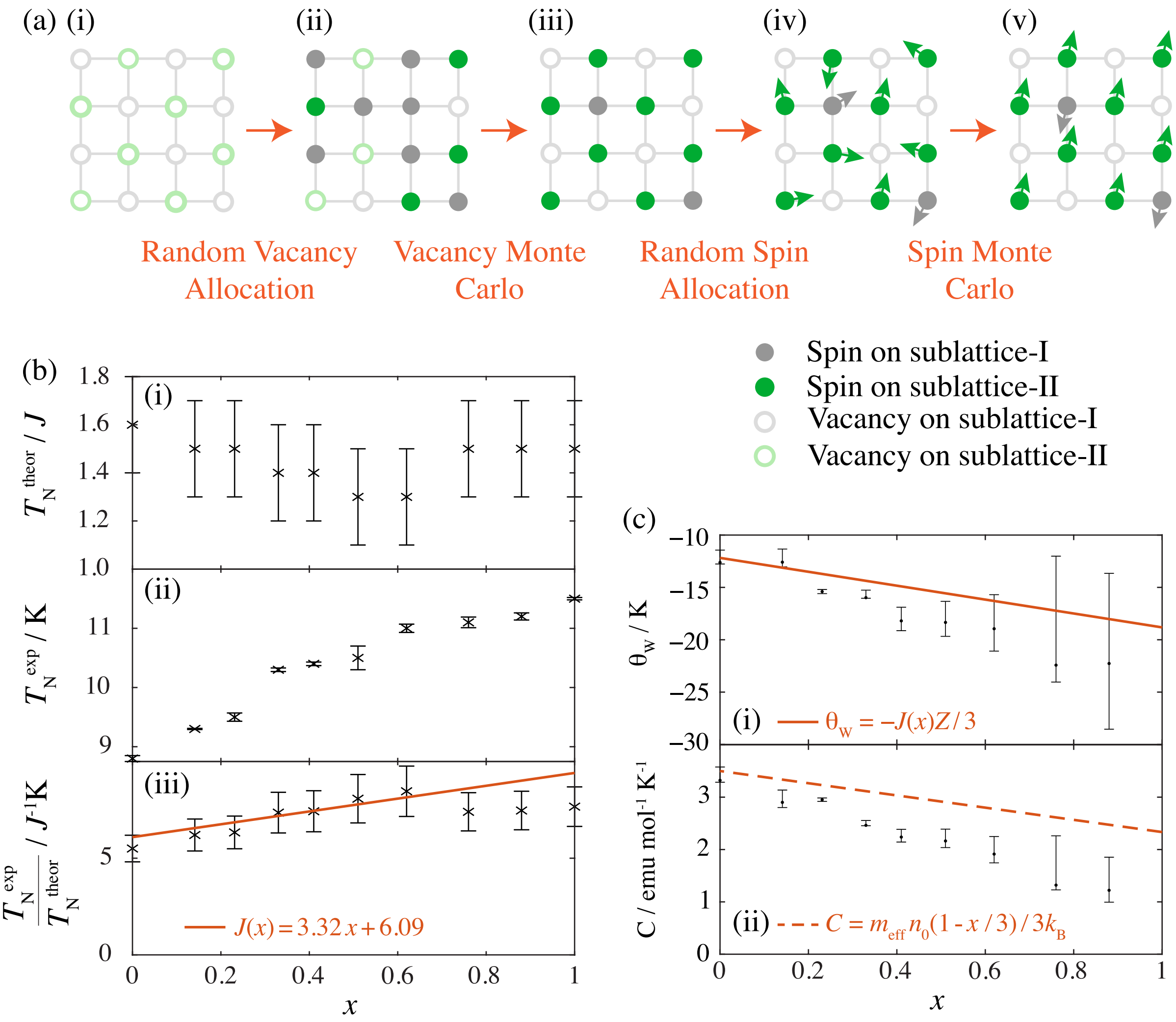

Figure S4: (a) Schematic showing the approach to MC simulations in these systems: (i) the bare lattice partitioned into an I and II sublattice, (ii) the system after randomly assigning the number of vacancies corresponding the stoichiometry x, (iii) the vacancy MC step minimises the number of nearest neighbour vacancy contacts, (iv) the system after decoration with Heisenberg spins, and (v) by the end of the spin Monte Carlo step at a low temperature, the system has ordered into a near-groundstate configuration. (b) The approach taken to parameterising $J(x)$ in Equation (5): (i) the theoretically determined transition temperature, (ii) the value experimentally derived from the divergence of ZFC and FC susceptibility, and (iii) their ration, shown alongside a linear fit. (c) The curie fit parameters (i) θ_{W} and (ii) C, from Fig. S2(b-c), shown alongside their values expected in the mean-field approximation for $J(x)$ given in Equation (5).

where N_{vac} is the number of nearest neighbours. This equation is normalised such that the value of $\alpha = 1$ would be produced by a chequerboard arrangement which involves the maximum possible order for a given concentration. Using this MC approach, we have reproduced the x dependence of the order parameter [Fig. 2(a) in the main text] in Ref. 1: the order parameter is low when $x < x_{\text{p}} = 0.6$ but increases rapidly at this percolation threshold. Although no long range order exists in the model for $x < x_{\text{p}}$, a statistically fluctuating imbalance between the occupation of sublattice I and II leads to some non-zero values for α. This finite size effect would disappear if the simulation was scaled up since the size of the fluctuations grows slower than the size of the system.

Spin MC

The next step of the modelling approach is to decorate the magnetic atoms in this model with spins. We have seen from the $x = 0$ endmember that the biggest term in the magnetic Hamiltonian is antiferromagnetic Heisenberg exchange. Any terms in the Hamiltonian besides Heisenberg exchange are neglected in our treatment. This approximation means that our model will be unable to reproduce the spin flop transition or residual magnetisation seen experimentally. However, we will go on to show that the model is able to capture the important PIF transition seen in this system. Based on these vacancy distributions ($o_i = 1, 0$ if occupied or unoccupied) we describe the dependence of the energy on the Heisenberg spin orientations $\mathbf{S}_j$ using the simplified ansatz Hamiltonian,

$$E_{\text{MC}} = J(x) \sum_{\langle ij \rangle} o_i o_j \mathbf{S}_i \cdot \mathbf{S}_j, \tag{5}$$

which again treats the three pseudocubic neighbours $\langle ij \rangle$ equivalently. Due to tilting patterns lowering symmetry, in $GuaMn(HCOO)_3$ there are two inequivalent neighbours which are bridged by anti-anti Mn–O–C–O–Mn exchange pathways. However, our approach, inspired by Ref. 4, treats these pathways as equivalent.

The MC simulation is carried out using a custom code related to that used in Ref.[S6]. This simulation takes configurations with frozen vacancy distributions generated by the previous MC step. The first series of simulations was carried out on the $6 \times 6 \times 6$ supercells. Simulated annealing is used, cooling from $T = 3J$ in steps of 0.05 K, halting after 100 h, typically at $T \approx 0.5J$. At each temperature, the algorithm measures decorrelation time n_{d}, equilibrates for $10n_{\text{d}}$, and progresses to measure 80 samples with $2n_{\text{d}}$ moves per sample. In this way, we ensure ergodicity in the simulations.

The net magnetisation of simulations at low temperature ($T = 0.5\,J$) is shown in Figure S4(bii). This

value tracks the order parameter quite well: increasing most significantly for $x \sim x_{\mathrm{p}}$ and staying quite low in the antiferromagnetic regime. An interesting observation is that the value increases with x even below x_{p} which may be consistent with the observation of some small ferrimagnetism below T_{C}. This may also be due to the same finite size effect noted earlier. Despite this, the result is clearly consistent with previous simulations of the PIF mechanism[S5] for this Ising model and extends these theories to the Heisenberg model.

Parameterisation

A common approach to parameterising such a Hamiltonian would be a fit to the low field magnetometry data. However, given the large errors in our values reported in Figure S1(b), this approach gives too large errors. Our approach (shown in Figure S4(b)) involves fitting to the value of the transition temperature, which has much smaller errors. The dependence of T_{N} on x is calculated from the model and experimentally, with a linear fit to their ratio taken as an estimate of $J(x)$. By this method, we obtain

$$J\,/\,\mathrm{K} = 3.32x + 6.09. \tag{6}$$

The $J = 6.09$ value for $x = 0$ falls within the range of values seen in other formate perovskites, e.g. between 5.7 and 6.5 K for the four systems studied in Ref. 4. The increase in J seen in its positive gradient could be for a variety of reasons. The increased charge from Mn^{2+} to Fe^{3+} could result in greater covalency interactions and therefore shorter bond lengths, both improving superexchange. Given the geometry-dependant magnetism seen as a function of pressure in related system[S7], the changing structure would likely result from relaxation around vacancies which may play a role too.

Simulating $\chi(T)$

Magnetic susceptibility is calculated from Monte Carlo simulations via the fluctuation method. The resulting values are plotted in Figure S5 and Figure 2(d) of the main text. In order to normalise for errors in measuring the mass of samples noted in section 2, values are normalised to their value at 15 K. This allows for direct comparison between MC and FC/ZFC results.

Note that the MC simulations give equilibrium values, which would be expected to lie between ZFC and FC curves (as indeed they generally do).

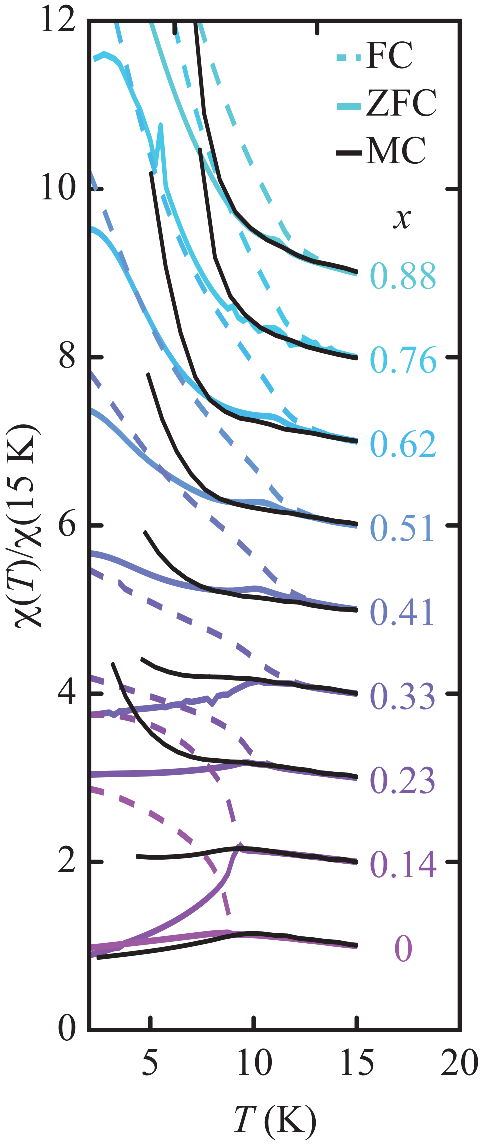

Figure S5: Field-cooled (dashed coloured lines) and zero-field-cooled (solid coloured lines) magnetic susceptibility ($H = 100$ Oe) as a function of temperature for nine members of the series [Gua]$Mn_{1-x}Fe_{2x/3}(HCOO)_3$ shown alongside the simulated values (solid black lines) generated from the MC procedure described in the text. To aid comparison, each data set is normalised to its value at 15 K and data for successive compositions are offset by one unit.

Domain formation

In order to investigate the role of domains, we move to even larger supercells: $20 \times 20 \times 20$. The simulations are carried out with the same approach as for the smaller supercell. However, it was not always possible to ensure decorrelation at the lowest temperatures so it became necessary to allow the simulation to proceed if not decorrelated within 10^4 moves per spin.

Single planes of the spin distribution at the lowest simulation temperature, $T = 0.2J$, are shown in Figure S4 for various values of x. In order to gain a little more insight from these configurations, it is easier to view local variations of the order parameter (again, $\mathbf{k} = \left[\frac{1}{2}, \frac{1}{2}, \frac{1}{2}\right]$) and magnetisation are plotted in Figure S4. These quantities are defined as

$$\Phi_{\text{loc.}}(\mathbf{R}) = \left[\sum_{i \in \text{sites}} w(|\mathbf{r}_i - \mathbf{R}|) o_i \exp 2\pi \mathrm{i} \mathbf{r}_i \cdot \mathbf{k}\right] \Big/ \left[\sum_{i \in \text{sites}} w(|\mathbf{r}_i - \mathbf{R}|)\right] \tag{7}$$

and

$$\mathbf{M}_{\text{loc.}} = \left[\sum_{i \in \text{sites}} w(|\mathbf{r}_i - \mathbf{R}|) o_i \mathbf{S}_i\right] \Big/ \left[\sum_{i \in \text{sites}} w(|\mathbf{r}_i - \mathbf{R}|)\right], \tag{8}$$

respectively. In this case, $\mathbf{R}$ is the position within the supercell, normalised so that the Mn–Mn separation is unity. These quantities allow us to see the spatial variation of the local order parameter and magnetisation in the neighbourhood of the spin at $\mathbf{r}$, defined an exponential window function,

$$w(\mathbf{r}) = \exp\left(-\frac{|\mathbf{r}|}{2\xi}\right), \tag{9}$$

where we use $\xi = 0.5$ unit cell distances.

Figure S6 displays the plot generated from Equation (7), where the red regions indicate the placement of vacancies on one fcc sub-lattice and the blue regions indicate their placement on the other fcc sub-lattice. For $x > x_{\mathrm{p}}$, we anticipated observing long-range order, which was confirmed by the macroscopic domains that spanned the entire structure. However, the domain size is similar to that of the supercell, indicating that finite size effects may still be present.

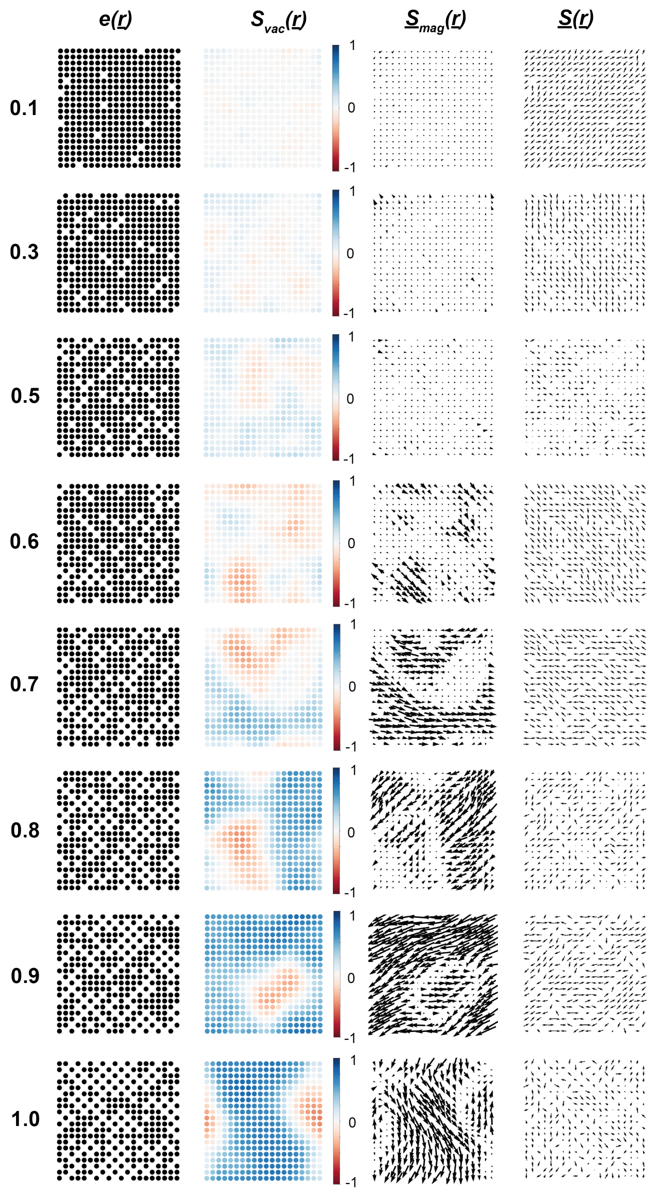

Figure S6: Vacancy and magnetic order plots over the range $0.1 < x < 1.0$. From left to right, the plots show the vacancy distribution ($e(\mathbf{r})$), the local vacancy order parameter ($S_{\mathrm{vac}}(\mathbf{r})$), the local magnetic order parameter ($S_{\mathrm{mag}}(\mathbf{r})$) and the vectors of the spins ($S(\mathbf{r})$). As x increases, the vacancy and magnetic order grows. The system percolates above $x = 0.6$ and results in a significant increase in vacancy order. As vacancies begin to preferentially occupy one fcc sub-lattice, this creates domains of checkerboard ordering. As a result the spin ordering becomes ferrimagnetic and a non-zero magnetic moment emerges.

4 References

(S1) H. L. B. Boström, J. Bruckmoser, A. L. Goodwin, *J. Am. Chem. Soc.* **141**, 17978 (2019).

(S2) S. Chen, R. Shang, K.-L. Hu, Z.-M. Wang, S. Gao, *Inorg. Chem. Front.* **1**, 83 (2014).

(S3) B. Liu, R. Shang, K.-L. Hu, Z.-M. Wang, S. Gao, *Inorg. Chem.* **51**, 13363 (2012).

(S4) W. Wang, L. Q. Yan, J. Z. Cong, Y. L. Zhao, F. Wang, S. P. Shen, T. Zou, D. Zhang, S. G. Wang, X. F. Han, Y. Sun, *Sci. Rep.* **3**, 2024 (2013).

(S5) P. Timonin, *Low Temp. Phys.* **40** (2013).

(S6) J. A. M. Paddison, J. R. Stewart, P. Manuel, P. Courtois, G. J. McIntyre, B. D. Rainford, and A. L. Goodwin, *Phys. Rev. Lett.* **110**, 267207 (2013).

(S7) I. E. Collings, R. S. Manna, A. A. Tsirlin, M. Bykov, E. Bykova, M. Hanfland, P. Gegenwart, S. van Smaalen, L. Dubrovinsky, N. Dubrovinskaia, *Phys. Chem. Chem. Phys.* **20**, 24465 (2018).
